# Human NKX2.2 influences islet endocrine cell fate choices through regulation of WNT pathway genes

**DOI:** 10.1101/2025.09.26.677825

**Published:** 2025-09-27

**Authors:** Christopher Schaaf, Fiona M. Docherty, Madison X. Rodriguez, Patrick Sean McGrath, Christopher J. Hill, Kristen L. Wells, Lori Sussel

**Affiliations:** 1 Barbara Davis Center for Diabetes, University of Colorado Anschutz Medical Campus, Aurora, CO, USA; 2 Present address: 1775 Aurora Ct, Aurora, CO, 80045, USA; 3 Senior author; 4 Lead contact

**Keywords:** NKX2.2, pancreatic endocrine progenitor cells, islet cells, transcriptional regulation, metabolic regulation

## Abstract

Transcriptional regulation is a key central mechanism of cell fate determination in developing tissues. The homeobox transcription factor NKX2.2 is an essential regulator of mouse and human pancreatic endocrine development, however its precise molecular role in a human system has not been previously investigated. In this study we generated NKX2.2 null (NKX2.2KO) human embryonic stem cell (hESC) lines using CRISPR/Cas9 technologies and differentiated them towards a pancreatic β cell fate using a stem cell-derived β cell differentiation protocol. Functional and transcriptomic analyses of the hESC-derived pancreatic endocrine cells lacking NKX2.2 revealed similarities and differences compared to the molecular functions of NKX2.2 in mice. In the absence of NKX2.2, the β cell differentiations result in reduced numbers of insulin-producing cells, and the differentiations become skewed towards polyhormonal fates, including cells co-expressing insulin, ghrelin and somatostatin. Deletion of NKX2.2 also eliminates the off-target formation of enterochromaffin cells. Single cell transcriptome analysis of the early endocrine cell population revealed a marked disruption of metabolic pathways that was confirmed by comparative metabolite tracing, providing novel insights into the regulation of early endocrine lineage decisions. Furthermore, NKX2.2 directly regulates several genes in the WNT signaling pathway, suggesting this is a key molecular mechanism through which NKX2.2 regulates these islet cell fate decisions in the human system.

## INTRODUCTION

In the developing mouse and human pancreas, a series of cell lineage decisions specify the distinct pancreatic cell types (reviewed in ^1,2^). A pancreatic progenitor (PP) pool is delineated by the expression of the transcriptional regulator PDX1, followed by the generation of tip cells marked by PTF1A, which ultimately give rise to the exocrine acinar tissue, or the bipotent progenitor trunk cells marked by SOX9. The bipotent progenitor population then gives rise to either ductal cells, marked by CDX2, or endocrine progenitor cells (EP) marked by NEUROG3. The EP cells give rise to the four distinct adult hormone-producing cell types: α cells (glucagon), β cells (insulin), δ cells (somatostatin) and PP cells (pancreatic polypeptide). These cells develop in small clusters within the pancreas, known as the islets of Langerhans. The tightly coordinated paracrine and endocrine actions of the islet α, β, and δ cells maintain proper glucose homeostasis; disruption of this process presents clinically as diabetes.

In mice, several studies have characterized the transcription factor (TF) homeobox protein NK-2 homolog B (NKX2.2) as a necessary factor for β cell development ^3–8^. NKX2.2 is expressed throughout the pancreas shortly after the onset of PDX1 expression, and then becomes restricted to the α, β and PP lineages ^9^. In mice deleted for *Nkx2.2* β cells are never generated, and there is a dramatic reduction in the number of α cells ^8^. Correspondingly, there is an increase in the number of ghrelin-producing ε cells ^10^. This defect is phenocopied in mice deleted for *Nkx2.2* specifically within the NEUROG3^+^ endocrine progenitor population ^3^. Molecular analyses have demonstrated that NKX2.2 binds and directly regulates the expression of target genes necessary for α and β cell fates ^6,11^. NKX2.2 has been shown to function in cell-type specific manners within the islet: in a β cell context it has been shown to form a repressor complex with DNMT3a to remodel chromatin and repress expression of α cell specific factors ^7^, and in an α cell context through recruitment of the α cell specific cofactor KLF4 ^11^. Together, these studies demonstrated that NKX2.2 plays a critical and necessary role in specifying endocrine cell fates. In humans, homozygous mutations in *NKX2.2* led to undetectable serum insulin levels at birth and neonatal diabetes, suggesting it has similar regulatory functions to those observed in mice ^12^. However, glucagon and ghrelin expression in these patients was not reported. Furthermore, immunofluorescence analyses of NKX2.2 expression in human fetal tissue suggested that, unlike in mice, NKX2.2 expression is activated later during pancreas development, downstream of NEUROG3 ^13^.

A promising approach to developing a functional cure for type 1 diabetes (T1D) is through the generation of stem cell-derived β-like cell differentiation protocols ^14^. These protocols use either human embryonic stem cells (hESCs) or human induced pluripotent stem cells (hiPSCs) to recapitulate human development and generate glucose responsive β-like cells. The ultimate goal is to use these β-like cells as a means for cell replacement therapies to unburden individuals with T1D from the need for constant management of the disease through blood glucose monitoring and exogenous insulin administration. In addition, the human stem cell model system provides a platform through which the investigation of the early stages of human pancreas development can be achieved, and molecular mechanisms can be investigated in full detail. In this study, we utilized *NKX2.2* knockout (NKX2.2KO) hESC lines to investigate the role of NKX2.2 in a human context. These studies suggest NKX2.2 is expressed concomitantly with NEUROG3 induction to regulate islet cell fate specification, although the phenotypes are somewhat different than those observed in mice lacking *Nkx2.2*. We also identify a unique role for NKX2.2 in regulating the WNT pathway and metabolic processes in the intermediate EP population to influence endocrine commitment.

## RESULTS

### NKX2.2 is concomitantly expressed with NEUROG3 during the in vitro generation of stem cell derived β-like cells.

**Figure 1.**
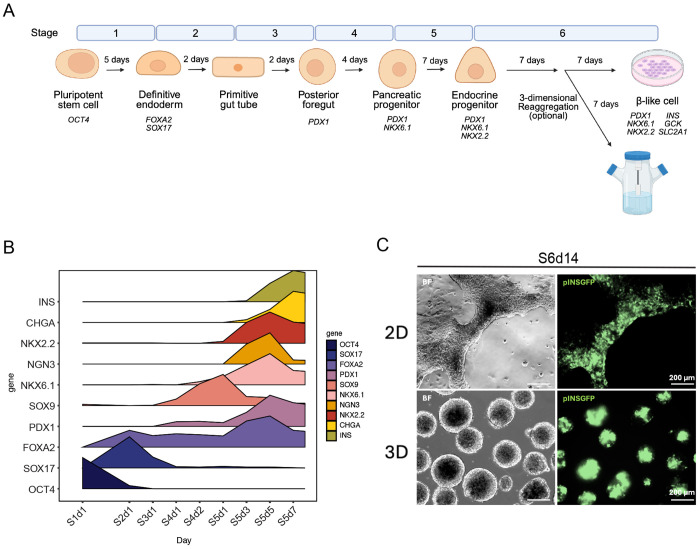
Differentiation protocol generates INS^+^ β–like cells. **A.** Schematic of 6-stage differentiation protocol with expected onset of gene expression in italics. **B.** qPCR time-course of critical endocrine development genes, data are presented as the mean expression relative to TBP, normalized to the maximum expression of the gene, scale 0-1 (data are representative of n = 3 independent differentiation). **C.** Imaging of end-stage (S6d14) WT β-like cells. Brightfield (BF) and GFP, indicating INS expression, images of cells differentiated in 2D and clusters that were reaggregated at S6d6 (scale bar = 200 μm).

To first clarify when NKX2.2 is expressed during the *in vitro* differentiation of hESC derived β-like cells, we differentiated the MEL-1 cell line expressing GFP from the INS promoter (MEL1 INS^GFP/w^, WT) ^15^ using the multi-step two-dimensional protocol developed by Hogrebe et al. ^16^ (Figure 1A). Quantitative PCR (qPCR) was used to monitor transcription factor expression at each critical stage of the differentiation protocol and identify when NKX2.2 was activated. This analysis confirmed the differentiation process proceeded as previously described and indicated that *NKX2.2* expression was induced in the endocrine progenitor (EP) cells expressing *NEUROG3* from stage 5 day 1 (S5d1), reaching a peak in expression at S5d4/5 and remaining activated for the duration of the differentiation (Figure 1B). Fluorescence microscopy analysis confirmed that these cells were successfully differentiated into β-like cells that robustly expressed GFP at the end stage of the 2D protocol (S6d14) and formed GFP-positive islet clusters upon 3D induction (Figure 1C).

### Loss of NKX2.2 results in a decrease in β-like cell numbers and the increased formation of polyhormonal cells.

**Figure 2.**
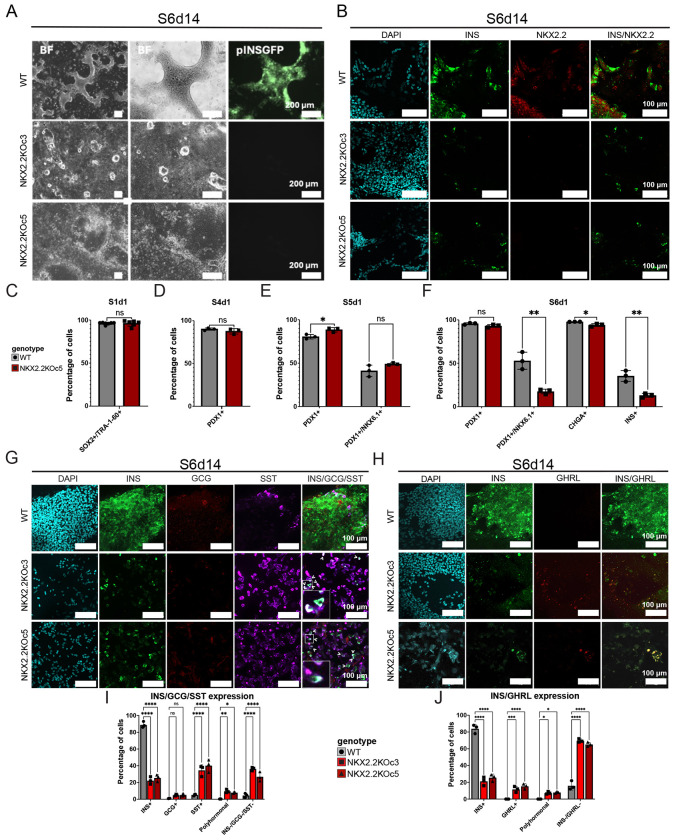
Loss of NKX2.2 results in a decrease in β-like cell numbers and the increased formation of polyhormonal cells. **A.** Imaging of end-stage (S6d14) WT and NKX2.2 KO β-like cells. Brightfield (BF) and GFP, indicating INS expression (scale bar = 200 μm) **B.** Immunofluorescent confocal imaging of insulin and NKX2.2 in WT and NKX2.2 KO β-like cells (scale bar = 100 μm) **C.** Quantification of flow cytometry analysis (WT n = 7, NKX2.2KOc5 n = 6) for pluripotency markers at the beginning of the differentiation (S1d1) **D-F.** Quantification of flow cytometry analysis (n = 3 independent differentiations each from WT and NKX2.2KOc5) at the end of the posterior foregut (S4d1) the end of the pancreatic progenitor (S5d1) and the end of the endocrine progenitor stages (S6d1). **G.** Immunofluorescent confocal imaging of insulin, glucagon, and somatostatin in WT and NKX2.2 KO β-like cells. Polyhormonal cells are marked by a caret in the merged image, and highlighted by the popout (scale bar = 100 μm) **H.** Immunofluorescent confocal imaging of insulin and ghrelin in WT and NKX2.2 KO β-like cells **I-J.** Quantification of hormone expression IF, represented as percentage of total cells. Three slides per condition. Two-way ANOVA (ns = not significant, * = p-val <0.05, ** = p-val <0.01, *** = p-val <0.0001, **** = p-val <0.00001. Student’s t-test).

To characterize the role of NKX2.2 in endocrine cells, we utilized a CRISPR/Cas9 targeting system to generate *NKX2.2* knockout (NKX2.2KO) hESC lines. A single guide RNA was used to target the first exon of *NKX2.2* in the WT parent cell line ^17^ which resulted in a homozygous single base-pair deletion at the gRNA cut site (Supplemental Figure 1A). This resulted in a frameshift mutation in the NKX2.2 protein and produced an early stop codon 29 amino acids downstream of the cut site (Supplemental Figure 1B). The genetic mutations were confirmed via Sanger sequencing (Supplemental Figure 1C - 1D). To assess the ability of the mutant clones to differentiate through the multistep protocol, WT and NKX2.2KO cells from two independent clones (c3 and c5) were guided through the 2D differentiation protocol ^16^ (Figure 1A). Bright field and fluorescence imaging demonstrated the ability of both clones to progress through the entire differentiation protocol; however, whereas the parent cell line showed robust GFP expression at the mature β-like cell stage (S6d14) GFP was undetectable in both NKX2.2KO clones (Figure 2A). Immunofluorescent (IF) and confocal imaging at the mature β-like cell stage (S6d14) confirmed a complete loss of the NKX2.2 protein in both clones and confirmed there was a substantial reduction in INS expression (Figure 2B).

### NKX2.2 regulates endocrine specification within the EP population

To identify the initial stages of differentiation at which NKX2.2 functions, we performed flow cytometry (FC) for known stage-specific markers throughout the differentiation process in the WT and NKX2.2KOc5 cell lines. FC for pluripotency markers SOX2 and TRA-1-60 showed that the NKX2.2KOc5 cell line maintained pluripotency after targeting (Figure 2C). At the pancreatic progenitor and EP stages (S4d1 and S5d1) there was no observed difference in the percentage of cells expressing PDX1 (Figure 2D) or co-expressing PDX1 and NKX6.1 (Figure 2E). However, by the end of the EP stage (S6d1), there is a marked difference between the WT and NKX2.2KO clones. While the PDX1^+^ population is unchanged in the NKX2.2KO cells, the PDX1^+^/NKX6.1^+^ is significantly decreased suggesting that in the absence of NKX2.2, NKX6.1 expression is not being maintained. By S6d1, fewer NKX2.2KOc5 cells express CHGA, although still the majority in both cell lines are CHGA^+^, suggesting that the cells are pursuing an endocrine lineage. However, in the NKX2.2KO cells we observe a significant decrease in the number of INS^+^ cells. (Figure 2F). Taken together, the FC analysis suggests that NKX2.2 is not required for the initial formation of PDX1^+^/NKX6.1^+^ pancreatic progenitors, but has essential functions at the EP stage to maintain the expression of NKX6.1^+^, and in specifying an INS^+^ β-like cell fate trajectory.

Mice deleted for *Nkx2.2* not only displayed a complete absence of insulin-producing β cells, but also a severe reduction in glucagon-expressing α cells and a substantial increase in ghrelin-expressing ε cells. Somatostatin expression and the number of δ cells were not altered. In humans, individuals with loss of function mutations in *NKX2.2* have no detectable C-peptide at birth and elevated serum ghrelin; glucagon and somatostatin expression were not assessed ^12^. To determine the hormone status of the cells differentiated from NKX2.2KO hESCs we performed IF for glucagon (GCG), somatostatin (SST) and ghrelin (GHRL) at the mature β-like cell stage (S6d14). Only a small number of GCG-expressing cells were detectable in both the WT parent cell line and each of the NKX2.2 KO clones, likely reflecting the bias of the differentiation protocol towards the β cell lineage (Figure 2C). Interestingly, however, there appeared to be a substantial increase in the number of SST^+^ cells in both NKX2.2 KO clones (Figure 2D). There was also an increase in the number of GHRL^+^ cells, although not to the same extent seen in mice lacking *Nkx2.2* (Figure 2D). We also observed the presence of polyhormonal cells – predominantly INS^+^/SST^+^ and INS^+^/GHRL^+^ co-expressing cells in the NKX2.2KO cell lines (Figure 2C–D). Cell quantification confirmed there was a significant reduction in monohormonal INS^+^ cells and a significant increase in SST ^+^**,** GHRL^+^ and polyhormonal cells. There were relatively equal numbers of a few GCG ^+^ cells in WT and both NKX2.2KO cell lines (Figure 2E).

### NKX2.2KO cells display disrupted endocrine cell specification downstream of NEUROG3.

**Figure 3.**
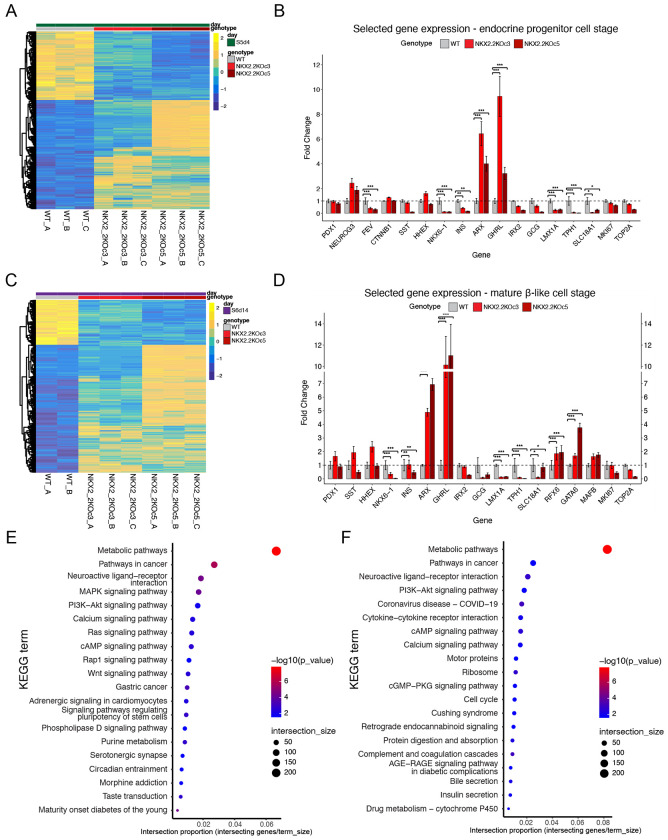
NKX2.2 KO cells display dysregulation at the endocrine progenitor stage. **A.** Differentially expressed genes (adjusted p-val <0.05) from bulk RNAseq samples (n=3 independent differentiations from each cell line) at the EP stage (S5d4). 3320 DE genes **B.** Fold change of selected genes from bulk RNAseq at the endocrine progenitor stage (* = p-val <0.05, ** = p-val <0.01, *** = p-val <0.0001. DESeq2 Wald tests with Benjamini-Hochberg correction) **C.** Differentially expressed genes (adjusted p-val <0.05) from bulk RNAseq samples (n = 3 independent differentiations from each cell line) at the mature β-like cell stage (S6d14). 2093 DE genes. **D.** Fold change of selected genes from bulk RNAseq at the mature β-like cell stage (* = p-val <0.05, ** = p-val <0.01, *** = p-val <0.0001. DESeq2 Wald tests with Benjamini-Hochberg correction). **E.** KEGG analysis of the differentially expressed genes at the EP stage (S5d4) **F.** KEGG analysis of the differentially expressed genes at the mature β-like cell stage (S6d14).

To characterize the underlying transcriptional changes that were contributing to the disrupted endocrine cell lineage specification, we performed bulk RNA sequencing (RNAseq) at the onset of endocrine cell differentiation (S5d4) from the WT and NKX2.2KO cell lines (n = 3 independent differentiations of WT, c3 and c5). Differential gene expression analysis revealed 3320 significantly differentially expressed (DE) genes in the NKX2.2KO cell lines (Figure 3A). Consistent with the FC analysis, *PDX1* expression was unaffected in the NKX2.2KO cell lines, and *NKX6.1* and *INS* expression were almost undetectable (Figure 3B). There was also a significant increase in expression of the *GHRL* hormone and the *ARX* transcription factor. The increased expression of *ARX* is likely associated with the increased number of ε cells, since there was no evidence of an increase in *GCG* expression or GCG expressing cells (Figure 3B and 2C). Correspondingly, the α cell transcription factor *IRX2* was also expressed at low, but unaffected levels. Also consistent with the expression of NKX2.2 during the differentiation process, there was no apparent effect on NEUROG3 expression. The significant down regulation of *FEV* expression, however, suggests that NKX2.2 functions downstream of NEUROG3 to regulate formation of at least a subset of intermediate EP cells ^1,14,18^. Interestingly, there was also a significant reduction in the expression of the enterochromaffin markers LMX1A, SLC18A1 and TPH1 suggesting that deletion of NKX2.2 suppresses the formation of this common off-target cell population. Similar relative changes in gene expression were maintained at the end stage of differentiation (S6d14), although moderate variability in gene expression levels between the two mutant clones began to emerge (Figure 3C–D).

### NKX2.2 regulates a number of metabolic and developmental signaling pathway genes

To gain a more comprehensive picture of the genetic pathways disrupted by the loss of NKX2.2, we performed KEGG analysis to identify significantly disrupted transcriptional pathways (Figure 3E–F). Interestingly, metabolic pathway genes represented the most significant changes in both the NKX2.2KO EP cells and β-like cells. Many important developmental signaling pathway genes were also mis-expressed, including those involved in MAPK and WNT signaling. Not surprisingly, given the significant reduction in β-like cells, there was also a significant reduction in genes regulating calcium signaling and insulin secretion.

### Loss of NKX2.2 disrupts β cell lineage commitment and signaling pathways within the endocrine progenitor population

**Figure 4.**
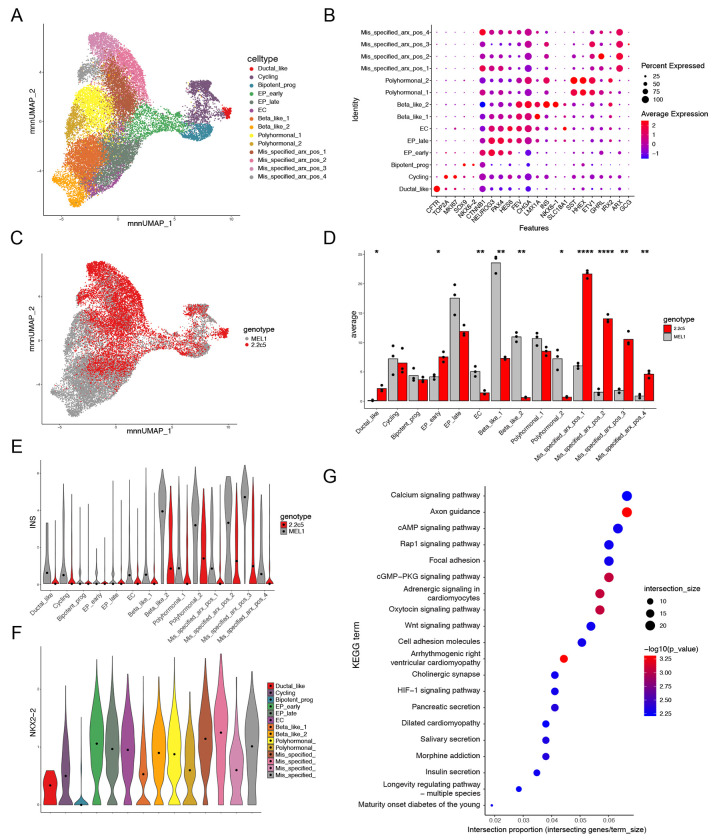
NKX2.2 regulates β cell lineage commitment and signaling pathways within the endocrine progenitor population. **A.** UMAP of subset of *PDX1*^+^ cells from the merged dataset of WT and NKX2.2KO differentiations at the mid-endocrine progenitor stage (S5d4, n=3 independent differentiations of each cell line) **B.** Gene expression of previously described cell type markers across clusters. **C.** UMAP colored by genotype **D.** Cell type cluster distribution by genotype represented as bar-chart. (* = p-val <0.05, ** = p-val <0.01, *** = p-val <0.0001. Student’s t-test). **E.** *INS* expression across clusters separated by genotype. Median expression of each group represented by dot. **F.** Expression of *NKX2.2* across clusters. Median expression marked by dot. **G.** Pathway overrepresentation analysis of the differentially expressed genes in the EP early cluster.

Because the β-like cell differentiation protocols are known to generate heterogeneous populations, we sought to identify which specific cell populations were dysregulated in the absence of NKX2.2. We performed single cell RNA-sequencing (scRNA-seq) on WT and NKX2.2KO cells (n = 3 differentiations per cell line) at S5d4, corresponding to the mid-EP stage where phenotypic differences were first observed. Cells were subset to *PDX1^+^* populations and annotated using previously described marker genes (Table 1, Figure 4A–B, Supplementary Figure 2). Remarkably, although both WT and NKX2.2KO cells were present in the bipotent progenitor and early/late EP populations, they diverged into two distinct branches (Figure 4C). Quantification revealed comparable numbers of WT and NKX2.2KO cells in the bipotent progenitor and EP late populations, with a modest increase in NKX2.2KO cells in the EP early population (p = 0.01, Figure 4D). Corresponding to what was observed in the bulk RNA-Seq data, the enterochromaffin cell population appears nearly absent in the NKX2.2KO samples (p = 0.04). Notably, β-like cells co-expressing *NKX6.1* and *INS* were significantly enriched in WT cells, whereas these cells were nearly absent in the NKX2.2KO samples (p = .001, Figure 4D). Instead, NKX2.2KO cells appeared to shift toward a mis-specified cell population co-expressing *ARX* and *INS* and lacking canonical β cell transcription factors. Although also present in the WT differentiations, the proportion of these mis-specified cells was significantly higher in the NKX2.2KO cells (p < 0.001, Figure 4D). Additionally, polyhormonal cells expressing multiple hormones – including *GHRL*, *SST*, and *INS* – along with transcription factors *HHEX* and *ETV*1 were identified in both genotypes (Figure 4D). Expression of *INS* was lower in the NKX2.2KO cells (Figure 4E) and fewer cells expressed *INS* (35% in NKX2.2KO vs 55% in WT). These findings suggest that while *INS*^+^ EP cells are generated in the NKX2.2KO cultures, they are fewer in number and either produce a mis-specified *ARX*^+^/*INS*^+^ population, are polyhormonal and/or lack key β cell transcriptional regulators.

To further investigate the transcriptional consequences of NKX2.2 loss, we utilized the single cell data to identify the expression dynamics of *NKX2.2* in WT cells (Figure 4F). Consistent with the qPCR expression data, *NKX2.2* expression was low in the bipotent progenitors, became upregulated in EP early cells and remained elevated throughout subsequent differentiation. Given that EP early cells are present in both genotypes, we assessed cell specific gene expression in this population. Differential gene expression analysis followed by pathway overrepresentation analysis (ORA) between WT and NKX2.2KO cells within the EP early population revealed dysregulation of several signaling pathways, including calcium, cAMP, and WNT, as well as secretion-related pathways such as pancreatic and insulin secretion (Figure 4G). These results complement the bulk sequencing data to confirm that NKX2.2 is required within the EP population to establish proper signaling and secretory programs essential for β cell function.

### NKX2.2 regulates glycolytic genes within the endocrine progenitor population

**Figure 5.**
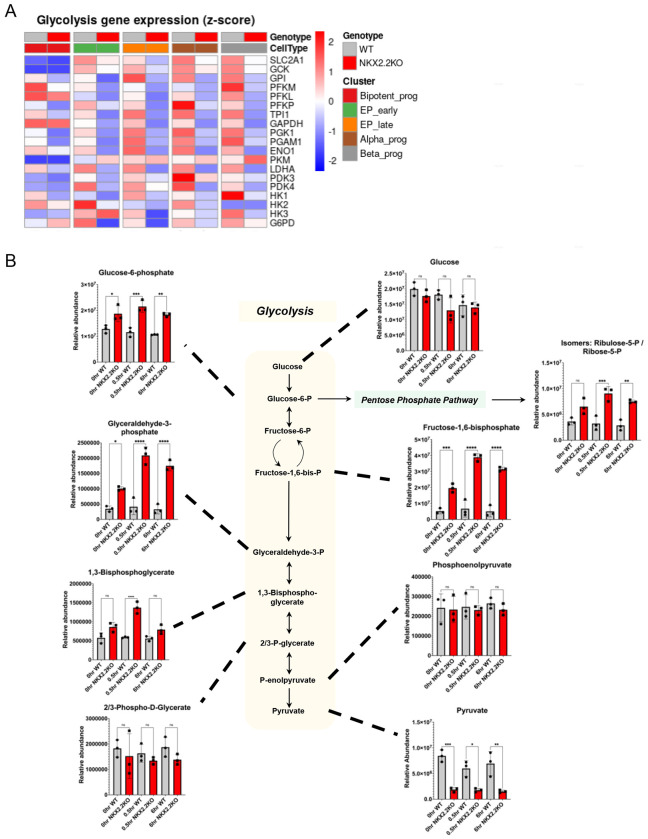
NKX2.2KO cells have dysregulated glycolytic gene expression and disrupted metabolite production. **A.** Heatmap of glycolysis pathway gene expression represented as a z-score across early endocrine progenitor subset clusters. **B.** Comparative global metabolite levels between WT and NKX2.2KO EP cells (S5d4, n=3 independent differentiations per cell line). Samples were collected at 0hr, 0.5hr, and 6hr timepoints. Student’s t-tests were performed between the WT and KO at each timepoint. (* = p-val <0.05, ** = p-val <0.01, *** = p-val <0.0001).

Bulk sequencing of the EP population revealed a significant disruption of metabolic pathway genes (Figure 3F) therefore we used the single cell sequencing data to identify the specific gene changes within each progenitor population. This analysis determined that many critical regulators of glycolysis were down regulated, including *GCK, PDK3, PDK4, PGK1, GPI, ENO1, GAPDH, ACLY* and *TPI1* (Figure 5A), suggesting that the NKX2.2KO EP cells were not appropriately processing metabolites through the glycolytic pathway. To characterize the metabolic implications of these gene expression changes, we performed a comparative global metabolite analysis in S5d4 WT and NKX2.2KO cells. Cells were incubated in ^13^C-labeled glucose containing media and both cells and supernatant were collected at 0 hours, 0.5 hours, and 6 hours timepoints. Cells from each time point were subjected to ultra-high performance liquid chromatography-mass spectrometry analysis with relative quantification (Figure 5B). At each timepoint, there were no observed differences in the total amount of cellular glucose, indicating that any metabolic defects were occurring internally, and not the result of deficient glucose import. Consistent with the gene expression analysis suggesting lack of NKX2.2 caused defects in glycolysis, we observed an upregulation of the glycolytic intermediates: glucose-6-phosphate, fructose-1,6-bisphosphate, glyceraldehyde-3-phosphate, and 1,3-bisphosphoglycerate at all timepoints in the NKX2.2KO EP cells, suggesting a block was occurring distally in the pathway (Figure 5B). Interestingly, there was a significant decrease in pyruvate levels across all time points. It also appeared that metabolites were being shunted into the pentose phosphate pathway, because there was an increase in the terminal product ribose-5-phosphate (Figure 5B). Taken together, this analysis demonstrates that the NKX2.2KO EP cells are failing to undergo normal glycolysis, which may contribute to their impaired differentiation potential.

### NKX2.2 directly inhibits the WNT/β-catenin pathway

**Figure 6.**
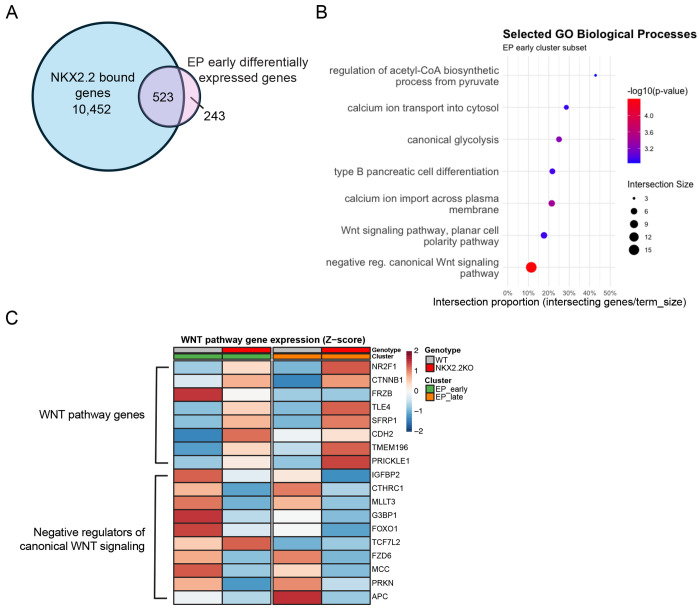
Dysregulation of WNT pathway genes in the NKX2.2KO early endocrine progenitors. **A.** Intersection of genes bound by NKX2.2 in ChIPseq of human islets and DE (NKX2.2KO vs WT) genes in the EP_early cluster. **B.** Selected GO Biological processes significantly overrepresented in the overlap of NKX2.2 human islet ChIPseq and DEGs from the EP_early cluster **C.** Heatmap of WNT/b-catenin pathway genes expression across the EP_early and EP_late clusters. Z-score is represented by color.

To begin to identify genes and pathways that are directly regulated by NKX2.2 to promote appropriate pancreatic endocrine lineage decisions, we re-analyzed a published NKX2.2 ChIP dataset from human islets ^19^ and intersected the data with the 766 DE genes between WT and the NKX2.2KO clones in the early EP cluster (Figure 4A). Remarkably, 68% (523/766) of the dysregulated genes were bound by NKX2.2 suggesting direct regulation (Figure 6A). Gene ontology analyses of the NKX2.2 bound and differentially expressed genes revealed significant enrichment for genes in a number of pathways including those associated with glycolysis, calcium signaling, β cell differentiation and most significantly, the WNT signaling pathway (Figure 6B). WNT signaling is known to be important for appropriate pancreas development, with downregulation of the pathway being a necessary step for endocrine commitment to proceed. Gene expression across pseudotime revealed that the β-catenin gene *CTNNB1* is significantly upregulated in the NKX2.2KO cells in the EP early cluster and has three NKX2.2 binding sites in its enhancer region (data not shown). Consistent with the upregulation of *CTNNB1*, there is downstream dysregulation of the WNT/β-catenin signaling pathway in the EP early and EP late clusters (Figure 6C), including the upregulation of *SFRP1, CDH2, TMEM196, PRICKLE1*, and *TCF7L2* and downregulation of negative WNT pathway regulators, *FRZB, IGFBP2, CTHRC1, MLLT3, G3BP1, FOXO1, FZD6, MCC*, and *PRKN*. This suggests that NKX2.2, through repression of *CTNNB1*, plays a critical role in the inhibition of the WNT/β-catenin pathway which is necessary for endocrine commitment to occur.

## DISCUSSION

The purpose of this study was to uncover the role of NKX2.2 in regulating the development of human pancreatic endocrine cells using an *in vitro* hESC-derived β cell differentiation platform. Homozygous hESC cell lines deleted for *NKX2.2* were guided through a β cell differentiation protocol^16^. The NKX2.2KO cells generated fewer INS-producing cells but had increased numbers of SST and GHRL expressing cells, in addition to polyhormonal INS^+^/SST^+^ and INS^+^/GHRL^+^ cells. There was also a significant increase in a population of mis-specified cells that expressed the *ARX* transcription factor and *INS*. IF, FC and transcriptome analyses demonstrated that the NKX2.2KO cells failed to properly progress through endocrine differentiation, failing to induce the expression of the intermediate EP marker *FEV* and to maintain expression of the essential β cell transcription factor NKX6.1. Transcriptomic data at the bulk and single cell level revealed that in the absence of NKX2.2, there was a disruption in the glycolytic pathway and a failure to downregulate WNT signaling in the EP population, two processes that could contribute to the impaired endocrine cell specification.

To characterize when NKX2.2 is required for human islet differentiation, we used the 2D differentiation protocol described by Hogrebe et al ^16^. Similar to published data, the MEL-1 cell line could be efficiently differentiated into INS-producing cells that express the essential β cell transcriptional regulator NKX6.1, produce very few GCG^+^ cells and ~5% SST^+^ cells. However, single cell analysis of the parent MEL-1 cell line at the EP stage also revealed populations of cells co-expressing *INS* and the α and ε transcription factor *ARX*, yet did not express β cell transcription factors or other endocrine hormones, including *GCG* and *GHRL*. These cells could represent populations that would normally be destined to become α or ε cells but are being redirected to a β-like cell fate by the biased β–like cell differentiation protocol. The significant increase in this population of progenitors in the absence of NKX2.2, could reflect NKX2.2’s normal function to repress the ARX^+^/GHRL^+^ lineage, since NKX2.2 has been shown to be essential to repress the differentiation of ε cells in mice. This is also consistent with the observed increased number of GHRL-expressing cells at the end stage (S6d14) of the protocol, however to a much lesser extent that was observed in mice – which may again reflect the differentiation protocol that has been designed to bias towards a β–like fate.

In this study, we demonstrated that *NKX2.2* is expressed simultaneously with *NEUROG3* in the EP population. This is a later developmental timepoint than observed in mice, where NKX2.2 is expressed in the pancreatic progenitor population. However, in both mouse and in the hESC β–like differentiation protocol, NKX2.2 functions within the EP population downstream of NEUROG3; NKX2.2KO EP cells fail to upregulate *FEV*, a marker of the intermediate progenitor population, and fail to maintain expression of *NKX6.1*, the key β cell transcription factor. Furthermore, bulk and single cell sequencing analysis at the EP stage demonstrated there was an upregulation of the WNT signaling pathway. Previous studies have shown that downregulation of WNT signaling is a necessary step for endocrine commitment to proceed ^20,21^. Single cell analysis revealed that the gene encoding β-catenin, *CTNNB1*, was upregulated in the EP population of the NKX2.2KO cells, and re-analysis of a previously published NKX2.2 ChIP-seq data set from human islets revealed several NKX2.2 binding sites within its enhancers ^19^ (data not shown). This suggests that NKX2.2 may be acting to inhibit the WNT/ β-catenin pathway within the EP population through direct repression of *CTNNB1*.

Gene expression analysis within the EP population also revealed a disruption of metabolic pathways. Subsequent glucose flux analysis suggested the absence of NKX2.2 resulted in defects in the glycolytic pathways that led to decreased pyruvate levels. A growing body of work has recently described the importance of strictly regulated cellular metabolism in differentiating cells ^22,23^. Iworima and colleagues recently showed that in islet-like cell differentiations there is high reliance on glycolysis from the pluripotent stage through the pancreatic progenitor stage, and that at the end stage of the differentiation there is a significant increase in oxidative phosphorylation ^24^. Historically this has been interpreted as being primarily related to the energetic demands of the cell. However, the interplay between metabolic intermediates and epigenetic regulation have come into focus in recent years^25^. Taken together, this could suggest that the metabolic switching between the pancreatic progenitor stage and the mature β-like cell stage is a critical regulatory timepoint for the differentiation of β–like cells. A higher resolution understanding of the metabolic switching that occurs during critical stages of the differentiation, such as endocrine specification, may reveal metabolic regulators of cell fate specification, allowing for the achievement of directed differentiations.

Here, we revealed a mechanism through which the transcription factor NKX2.2 regulates endocrine specification transcriptionally and metabolically. By integrating a more holistic approach to differentiation protocols, where we account for the metabolic needs of differentiating cells as an additional mechanism for regulating cell fate decisions, we may be able to achieve the differentiation of cells that truly recapitulate mature, adult cell types.

## METHODS

### Maintenance and differentiation of MEL-1 hESCs

MEL-1 INS^GFP/w^ (RRID:CVCL_XA16) were maintained as described by Hogrebe et al.^16^ Briefly, hESCs were seeded onto Cultrex (R&D Systems, 3434-005-02) coated cell culture plates (Greiner, 07-000-208) in mTeSR Plus media (Stem Cell Technologies, 100-0276), and passaged every 3 to 5 days. Aggregate passaging was performed by incubation in 0.5 mM EDTA for 10 minutes at 37C, and the media was supplemented with 10 μM y-27632 (Tocris, 1254) for the first day following each passage.

MEL-1 INS^GFP/w^ hESCs were differentiated into mature islet-like cells and/or endocrine progenitors using the protocol described by Hogrebe et al.^16^ with some modifications. Single cell dissociation of 90% confluent hESCs was performed by enzymatic digestion with TrypLE Express (Gibco, 12604021). Cells were then suspended with mTeSR Plus media with 10 μM y-27632 and plated in Cultrex treated cell culture plates at a concentration of 3.125 x 10^5^ cells/cm^2^ for 24 hours. Cells were then washed with 1x PBS and then differentiated using a six-stage protocol with stage-specific medium. Medium was changed daily throughout the protocol until day 21, from which media was changed every other day.

Base medium for all stage-specific media was comprised of MCDB 131 medium (Thermo Fisher Scientific, 10372019) supplemented with D-glucose (Sigma-Aldrich, G8270), NaHCO3 (Sigma-Aldrich, S8875-500G), Glutamax (Thermo Fisher Scientific, 35050061), BSA (Sigma-Aldrich, A7030), and penicillin-Streptomycin (Thermo Fisher Scientific, 15140122) at the following concentrations:

- Stage 1 base media: MCDB 131 medium, 9 mM D-glucose, 0.1% BSA, 1% Glutamax, 1% Pen/strep, 14 mM NaHCO3
- Stage 2 base media: MCDB 131 medium, 4.5 mM D-glucose, 0.1% BSA, 1% Glutamax, 1% Pen/strep, 14 mM NaHCO3
- Stage 3/4 base media: MCDB 131 medium, 2.4 mM D-glucose, 2% BSA, 1% Glutamax, 1% Pen/strep, 21 mM NaHCO3
- Stage 5 base media: MCDB 131 medium, 20 mM D-glucose, 2% BSA, 1% Glutamax, 1% Pen/strep, 21 mM NaHCO3
- Stage 6 base media: MCDB 131 medium, 2.4 mM D-glucose, 2% BSA, 1% Glutamax, 1% Pen/strep

Media compositions for each stage were as follows:

- Stage 1 (days 1-4): base medium, 200 ng/mL Activin A (R&D Systems, 338-AC-01M), 3 μM CHIR99021 (Stem Cell Technologies, 72054 , only on day 1)
- Stage 2 (days 5-6): base medium, 50 ng/mL KGF (R&D Systems, 251-KG-01M), 2.5 μM L-ascorbic acid (Millipore Sigma, A4544)
- Stage 3 (days 7-8): 2 μM retinoic acid (Selleckchem, S1653), 50 ng/mL KGF, 0.25 μM SANT1 (Tocris, 1974/10), 0.2 μM LDN (Tocris, 6053/50), 0.2 μM TPPB (Tocris, 0761/50), 2.5 μM L-ascorbic acid, 0.5% ITS-X (Gibco, 51500-056)
- Stage 4 (days 9-12): 0.1 μM retinoic acid, 50 ng/mL KGF, 0.25 μM SANT1, 0.2 μM LDN, 0.2 μM TPPB, 2.5 μM L-ascorbic acid, 0.5% ITS-X
- Stage 5 (days 13-19): 2 μM retinoic acid, 0.25 μM SANT1, 1 μM L-3,3′,5-Triiodothyronine (Cayman Chemical, 30996), 1 μM γ-secretase-inhibitor (Millipore Sigma, 565790), 10 μM Alk5i II (ENZO Life Sciences, ALX-270-445-M005), 2.5 μM L-ascorbic acid, 0.5% ITS-X, 10 mg/L heparin (Millipore Sigma, H3149-50KU), 1 μM Latrunculin A (Cayman Chemical, 10010630, day 13 only)
- Stage 6 (days 20-34): base medium, 10 mg/L heparin, 1% MEM non-essential amino acids (Gibco, 11140050), 0.1% Trace elements A (Corning, 25-021-CI), 0.1% Trace elements B (Corning, 25-022-CI), 1μM ZnSO4 septa-hydrate (Millipore Sigma, 108883)

### Generation of NKX2.2 KO MEL-1 INS^GFP/w^ hESC lines

NKX2.2KO cell lines were generated at the Disease Modeling Core, Barbara Davis Center for Diabetes. To generate homozygous *NKX2.2* deletion MEL-1 INS^GFP/w^ cell lines, a CRISPR/Cas9 strategy was used with a sgRNA targeting the first exon of *NKX2.2*. To generate a pool of potential clones, the sgRNAs were introduced into MEL-1 INS^GFP/w^ hESCs using nucleofection. The modified clones were plated into mTeSR Plus medium supplemented with 10 μM y-27632 and 1 μg/mL puromycin for 72hrs. Individual colonies that emerged were then manually transferred into 48-well plates for clonal expansion. Clonal screening was performed by genomic DNA extraction and Sanger sequencing. Two clones with a single base-pair deletion in the first exon of *NKX2.2* were selected. Chromosomal integrity was confirmed via karyotyping analysis, performed by the University of Colorado Cancer Center Molecular Pathology Shared Resource.

### Immunofluorescent analysis

Cells were differentiated on circular glass coverslips. At the desired timepoint, media was aspirated, cells were washed with PBS and fixed with 4% paraformaldehyde for 30 minutes at room temperature. PFA was removed, wells were washed once with PBS, and ICC solution (0.1% Triton X and 5% normal donkey serum in PBS) was added to the wells for 45 minutes at RT to block and permeabilize the cells. Primary antibodies (Supplement Table S.1) were diluted in ICC solution and were added to the wells for overnight incubation at 4C. The following day, primary antibody solution was removed, wells were washed once with ICC solution, and wells were incubated in secondary antibodies diluted in ICC solution at 1:300 for 2 hours at RT, light protected. Secondary antibodies were removed, wells were washed once with ICC solution, and DAPI nuclear stain (1:1000 in ICC) was applied for 12 minutes at RT, light protected. DAPI solution was removed, wells were washed once with ICC solution, then once with PBS, and then the glass coverslips were mounted on microscope slides with ProLong Gold Antifade Mountant (Invitrogen, P36934). Images were obtained using a Zeiss LSM 800 Confocal Laser Scanning Microscope and analyzed with Fiji (ImageJ Ver 1.54p).

### Bulk RNA sequencing (bulk RNAseq) and differential expression analysis

#### Bulk RNA sequencing (bulk RNAseq) and differential expression analysis

Cells were differentiated to the desired timepoint, growth medium was aspirated, wells were washed once with PBS, and then digested with TrypLE for 6 minutes at 37C. Cells were collected from the wells and transferred into a 1.5 mL microcentrifuge tube, and centrifuged for 3 minutes at 300 x g. Cells were washed once with PBS, centrifuged for 3 minutes at 300 x g, resuspended in RLT Plus buffer, and immediately frozen at −80C. RNA isolations were then performed using RNeasy Plus Mini Kit (Qiagen, 74134). RNA was submitted to the CU Anschutz Genomics Shared Resource for library preparation and NovaSeq X sequencing. Paired-end library preparation of RNA samples containing a total amount of >500 ng was generated by the core using the Tecan Universal Plus mRNA-seq library preparation kit with NuQuant. All RNA-seq libraries were run on an Illumina NovaSEQ 6000 or NovaSEQ X aiming for 40 million read pairs/80 million total reads per sample. Fastq output files were run through FastQC (v0.12.1) for quality control, and adapters were trimmed from files using cutadapt (v4.8). Alignment was performed using STAR (2.7.11b) to the hg38 genome before creating a counts table with the featureCounts function from subread (v2.0.6). DGE analysis was performed on RNA counts with experiments containing at least a sample size of three per condition using DESeq2 in R (Version 1.46.0). Significant gene expression changes for all DGE analyses were determined with significance cutoff of ≤0.05. gprofiler2 (v0.2.3) was used for ORA analysis.

### Flow cytometry analysis

Cells were differentiated to the desired timepoint, removed from the wells by enzymatic digestion with TrpyLE Express for 7 minutes at 37C, washed with PBS, and fixed in 4% paraformaldehyde for 30 minutes at 4C. Cells were washed in PBS, and incubated in ICC solution for 12 minutes at RT. Conjugated antibodies were diluted in CAS-Block supplemented with 0.4% Triton-X (CAS-T block), added to the cells, and incubated overnight at 4C, light protected. The following day, cells were washed with ICC solution, resuspended in 200 μL of ICC solution, and filtered through the cap of a FACS tube. 25,000 suspended cells per sample were then analyzed in a Cytek Aurora 5L Spectral Flow Cytometer. Data was processed using FlowJo software version 10.10.0.

### Single cell RNA sequencing (scRNAseq)

Cells were differentiated to S5d4, culture medium was aspirated, cells were washed with PBS, and then single cell suspensions were generated by TrypLE digestion for 8 minutes at 37C. Cells were centrifuged at 300 x g for 5 minutes. The supernatant was removed, and cells were washed twice with 1 mL of PBS supplemented with 0.1% BSA and then placed on ice and delivered to the CU Anschutz Genomics Shared Resource for cell capture and sequencing. A total of 67,252 cells were captured. Over 10,000 cells per sample were loaded onto a 10X Chromium controller for GEM formation and barcoding using Chromium GEM-X Single Cell 3′ kit with v3 reagents. cDNA synthesis and library preparation were performed using 10X GEM-X Single Cell 3′ kit with v3 reagents according to the manufacturer’s instructions. Library quality control was performed using a Tapestation (High Sensitivity D1000, Agilent). Libraries were sequenced on a NovaSeq X sequencer (Illumina).

### Metabolomics

Cells were differentiated to S5d4, and media changes were performed with ^13^C-labeled glucose containing media. Cells and supernatant were collected at 0 hours, 0.5 hours, and 6 hours timepoints. The media used for the 0 hour timepoint did not contain ^13^C-labeled glucose, and served as a negative control for the glucose tracing. Cell and supernatant collections were performed by collection of 1 mL of conditioned media, which was flash frozen in liquid nitrogen and stored at −80C. Cell collection was performed by enzymatically digesting the cells with TrypLE Express for 8 minutes at 37C. Cells were collected, counted, TrypLE and quenching media was aspirated, and cell pellets were washed three times with ice-cold PBS. All supernatant was aspirated, and cells pellets were snap frozen in liquid nitrogen before being stored at −80C.

Relative metabolite quantification was performed by the University of Colorado School of Medicine Metabolomics Core (Aurora, CO). Metabolites were extracted by the addition of chilled (4°C) lysis buffer (LB) (5:3:2 methanol:acetonitrile:water (v/v/v)) to a concentration of 2 x 10^6^ cells/mL (based on approximate cell counts) to frozen cell pellets. Supernatant samples were extracted at a ratio of 1:25 (v/v) with the lysis buffer. Samples were subsequently vortexed for 30min at 4°C and centrifuged for 10min (18,000g, 4°C) to re-pellet the cells. The supernatant was then removed and placed in autosampler vials for UHPLC-MS analysis. A Thermo Vanquish UHPLC system coupled to a Thermo Orbitrap Exploris 120 mass spectrometer (Thermo Fisher Scientific) was used for untargeted metabolite analysis. Samples were analyzed both in positive and negative ion mode in separate runs using a 5min gradient method^26^.

Mass spectra peaks corresponding to the heavy (13C-labled) and light version of each metabolite were identified, quantified (peak area) and annotated using El-Maven v0.12.0^27^ Statistical analysis, pair-wise comparisons and figure generation were accomplished using Graphpad Prism 10.2.3.

### ChIP-seq data analysis

Publicly available human islet NKX2.2 ChIPseq peak files were obtained from ArrayExpress (Accession num. E-MTAB-1919). ChIP peaks were annotated using the ChIPseeker software (version 1.42.1). Peaks from both datasets were subset by overlapping peaks, with a maximum gap of 10 bp.

### scRNAseq raw data processing and data analysis

All packages and versions used in these analyses can be found in the docker image at hub.docker.com/r/kwellswrasman/scrna_seq_r:v3. R version 4.2.3 was used for all scRNAseq analysis unless otherwise noted.

#### Data processing using Cell Ranger Software

Alignment to the hg38 genome and initial processing were performed using the 10x Genomics Cell Ranger v8.0.0 pipeline. Each sample was processed individually prior to downstream analysis. Raw data generated by Cellranger were read into Seurat (v4.1.3)

#### Filtering of low-quality cells and doublets

Seurat objects were created for each sample. To filter cells with low quality RNA, each object was filtered for number of features, number of counts, and percentage of reads mapped to mitochondrial DNA using the perCellQCFilters and perCellQCFilters functions from the scuttle r package (v1.8.4). Doublets were identified and removed using the DoubletFinder package (version 2.0.3).

#### Cell clustering

After cell filtering, we performed PCA and extracted the top 30 principal components to calculate the 30 nearest neighbors, which were then used for UMAP dimensional reductions with a resolution of 0.3 and a min_dist = 0.3. All experimental Seurat objects were then merged into a single Seurat object. The same processing was performed with the following exceptions: top 50 principal components were extracted, UMAP dimensionality reduction was performed with top 36 PCs. Batch correction was performed on the merged object using mutual nearest neighbor (MNN) matching. Differential expression analysis of all clusters was performed by using the Seurat FindAllMarkers function to identify significantly enriched gene expression unique to each cluster. Cell cluster annotation was performed by identifying significantly enriched expression of known cell population markers in each cluster (Figure 4A, 4C). Unpaired t-tests were used to compare cell type proportions between genotypes. To determine the number of insulin positive cells, an expression cutoff of 0.5 was used in the normalized data. To identify differential genes within the EP early population, we used Seurat’s FindMarkers function to compare the genotypes only within the EP early cells. gprofiler2 (v0.2.3) was used for ORA analysis.

#### Pseudotime analysis

Pseudotime analysis was performed using the Slingshot package version 2.14.0 and R version 4.4.2. the ‘slingshot’ function was performed on the endocrine progenitor subset Seurat object using the PCA reduction. Lineages were set to begin at the bipotent-progenitor cluster, and end at either the EP_multipotent or EP_ α cluster. Pseudotime values across each lineage were extracted using ‘slingPseudotime’ and were then plotted onto the UMAP. Gene expression across pseudotime analysis was generated by extracting expression levels and pseudotime values from the Seurat object.

#### Gene ontology enrichment analysis

We performed gene ontology enrichment analysis of biological processes using the geneontology.org web-based tool. PANTHER overrepresentation test was performed using the Gene Ontology database (released 2025-03-16). Fisher’s Exact statistical test was performed and false discovery rates (FDR) were calculated. Only GO terms with a FDR P-value < 0.05 were reported.

#### Hypergeometric testing

Gene set enrichment analysis was performed using hypergeometric testing to identify overrepresented cell type signatures within the differentially expressed genes of each cluster. Cell type specific gene sets for human α, β, δ, and ε were obtained from Muraro, et al.^28^.

### Quantification and statistical analyses

Statistical analyses were performed using GraphPad Prism (v10.1.1 (270)) and R (v4.2.3 and v4.4.2, as indicated). Statistical parameters and test used are reported in the figures and figure legends. For all hESC experiments, “n” refers to the number of independent hESC differentiation experiments analyzed (biological replicates). All bar graphs are displayed as mean ± standard deviation (SD). Unpaired student’s t-tests were performed for two-sample observations. For multi-sample comparisons, one-way ANOVA was used.

## Supplementary Material

1

## Figures and Tables

**Table 1.** Single Cell Sequencing Cell Type Identification

Cell type	Genes	References
Bipotent progenitor	SOX9	^29^
EP early	NEUROG3, PAX4, CTNNB1, HES6	^20,30,31^
EP late	NEUROG3, PAX4, FEV, HES6	^18,31^
Beta like 1	FEV, CHGA, LMX1A	18
Beta like 2	INS, CHGA, NKX6-1, FEV, LMX1A,	^18,31^
Mis-specified beta like Arx+ 1	ARX, CTNNB1, GHRL, NEUROG3	^31^
Mis-specified beta like Arx+ 2	GHRL, ARX, ETV1, INS	^31^
Mis-specified beta like Arx+ 3	ARX, ETV1, INS, GCG	^31^
Mis-specified beta like Arx+ 4	ARX, CTNNB1, CHGA	^31^
Polyhormonal 1	SST, HHEX, ETV1, GHRL	^31^
Polyhormonal 2	SST, HHEX, INS, ETV1, CHGA, IRX2	^31^
EC	LMX1A, SLC18A1, TPH1	^32,33^
Ductal like	CFTR	^34^
Cycling	TOP2A, MKI67	

**Table 2.** List of antibodies used

Target	Host species	Experiment/dilution	Manufacturer	Catalog number
CHGA	Mouse	FC 1:50	Novus	NBP2-32956
GCG	Rabbit	IF 1:200	Cell Signaling	2760S
GHRL	Rabbit	IF 1:200	Phoenix	H-031-30
INS	Guinea pig	IF 1:10	DAKO	IR00261-2
INS	Rabbit	FC 1:50	Cell Signaling	9016S
Ki67	Rabbit	IF 1:500	Abcam	ab15580
NEUROD1	Mouse	FC 1:50	Bioss	bs-1517R-A350
NKX2.2	Mouse	IF 1:50	DSHB	74.5A5
NKX6.1	Mouse	FC 1:50	BD	563338
PDX1	Mouse	FC 1:20	BD	562161
PDX1	Goat	IF 1:200	R and D	AF-2419-SP
PPY	Goat	IF 1:200	Novus	NB100-1793
SST	Mouse	IF 1:100	BD Biosciences	566031
