# Supplementary material for: Human NKX2.2 influences islet endocrine cell fate choices through regulation of WNT pathway genes
Source: bioRxiv. 2025 Sep 27:2025.09.26.677825. Preprint. [Version 1] doi: 10.1101/2025.09.26.677825 (PMC12485700; doi:10.1101/2025.09.26.677825)
Supplement: 1 [file NIHPP2025.09.26.677825v1-supplement-1.pdf]

bioRxiv preprint doi: https://doi.org/10.1101/2025.09.26.677825; this version posted September 28, 2025. The copyright holder for this preprint (which was not certified by peer review) is the author/funder, who has granted bioRxiv a license to display the preprint in perpetuity. It is made available under aCC-BY 4.0 International license.

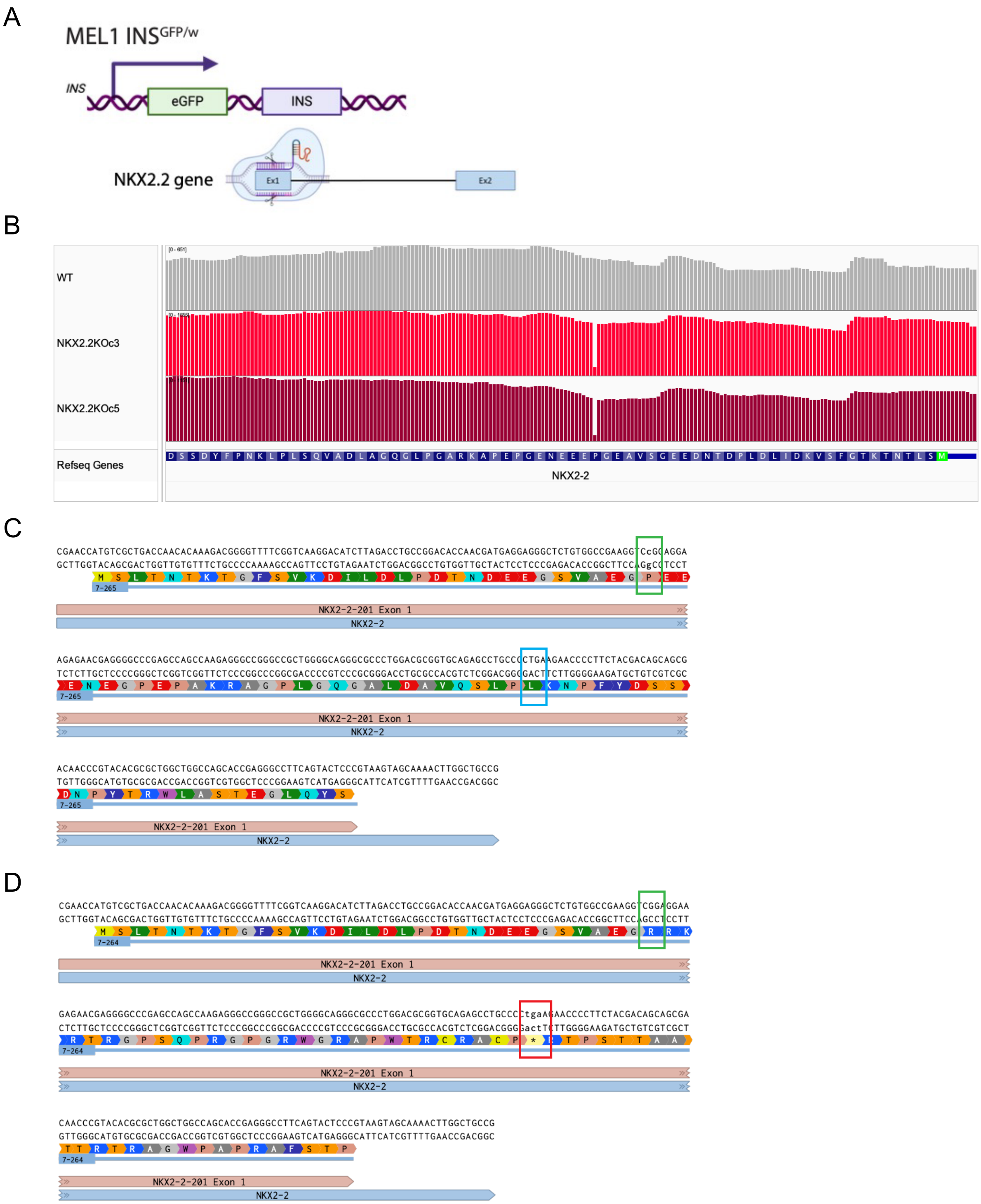

Supplemental figure 1. NKX2.2KO targeting strategy and validation.

bioRxiv preprint doi: https://doi.org/10.1101/2025.09.26.677825; this version posted September 28, 2025. The copyright holder for this preprint (which was not certified by peer review) is the author/funder, who has granted bioRxiv a license to display the preprint in perpetuity. It is made available under aCC-BY 4.0 International license.

A. Schematic of NKX2.2KO targeting strategy in the MEL1 $INS^{GFP/w}$ parent cell line. **B.** IGV tracks of bulk RNAseq data focused on the NKX2.2 loci. Absence of a single base pair is observed in both KO clones. **C.** Genomic sequence of the first exon of NKX2.2 with amino acid translations. Green box indicates the target cut site of the gRNA. Blue box indicates where the early stop codon appears. **D.** Genomic sequence representing the NKX2.2KO clones with deletion. Orange box identifies the absence of the cytosine and where the frameshift is induced. The red box identifies the early stop codon.

bioRxiv preprint doi: https://doi.org/10.1101/2025.09.26.677825; this version posted September 28, 2025. The copyright holder for this preprint (which was not certified by peer review) is the author/funder, who has granted bioRxiv a license to display the preprint in perpetuity. It is made available under aCC-BY 4.0 International license.

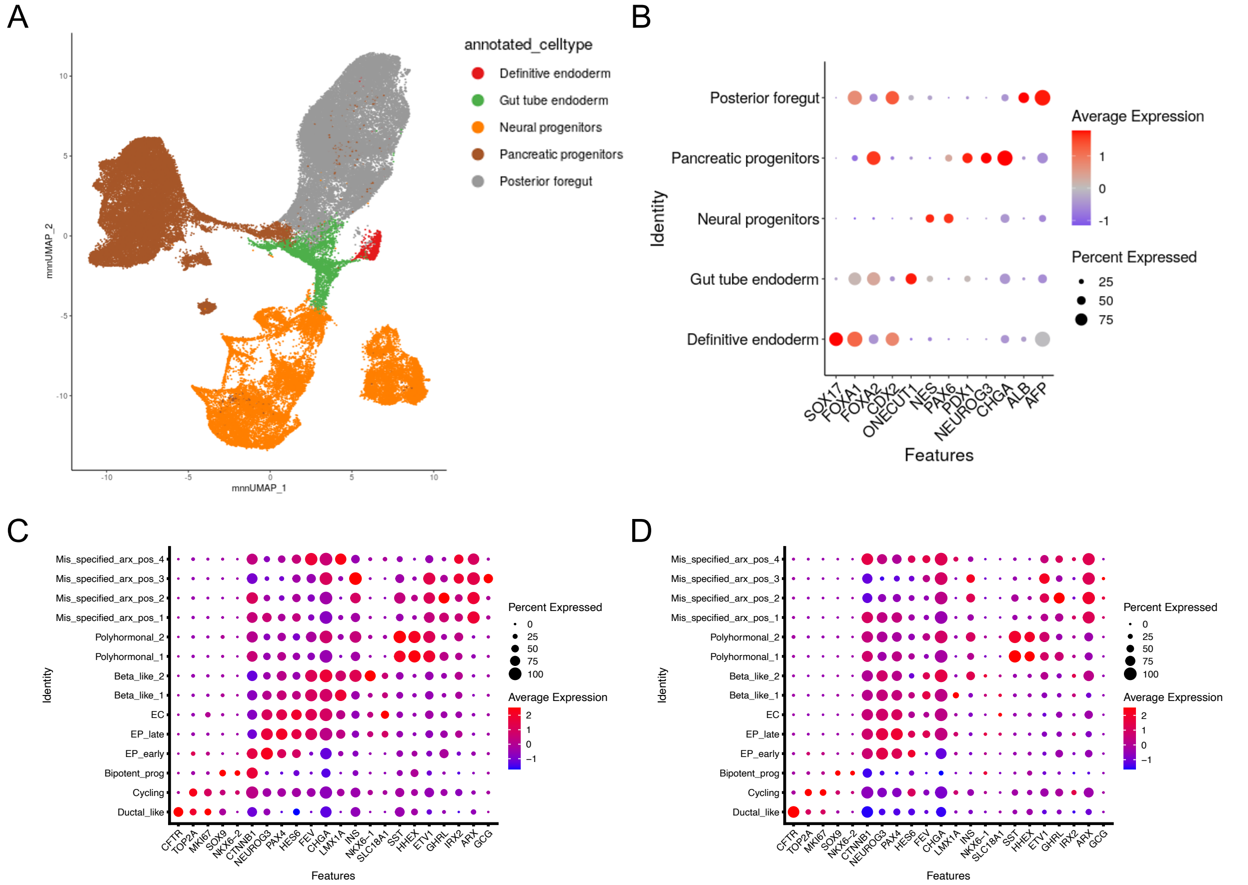

Supplemental figure 2. Cluster annotation for full scRNAseq dataset. A. UMAP of batch corrected, merged dataset containing all samples (n=3 independent differentiations each of NKX2.2KO and WT cell lines at S5d4). **B.** Cluster annotation performed based on expression of known markers of progenitor cell types. **C.** Gene expression of canonical cell type markers across clusters in the WT cell line. **D.** Gene expression of canonical cell type markers across clusters in the NKX2.2KO cell line.
